# Co-occurrence of pain and dyspnea in Veterans with COPD: Relationship to functional status and a pilot study of neural correlates using structural and functional magnetic resonance imaging

**DOI:** 10.1371/journal.pone.0254653

**Published:** 2021-07-15

**Authors:** Marilyn L. Moy, Rinu A. Daniel, Paola N. Cruz Rivera, Maria A. Mongiardo, Rebekah L. Goldstein, Diana M. Higgins, David H. Salat

**Affiliations:** 1 Pulmonary, Critical Care, and Sleep Medicine Service, VA Boston Healthcare System, Boston, MA, United States of America; 2 Harvard Medical School, Boston, MA, United States of America; 3 Boston University School of Medicine, Boston, MA, United States of America; 4 Anesthesiology, Critical Care, and Pain Medicine Service, VA Boston Healthcare System, Boston, MA, United States of America; 5 Neuroimaging Research for Veterans Center, VA Boston Healthcare System, Boston, MA, United States of America; 6 Athinoula A. Martinos Center for Biomedical Imaging, Massachusetts General Hospital, Boston, MA, United States of America; Prince Sattam Bin Abdulaziz University, College of Applied Medical Sciences, SAUDI ARABIA

## Abstract

Persons with COPD experience co-occurring dyspnea and pain. Little is known about the relationship between symptom co-occurrence with physical activity (PA) and exercise. Novel diagnostic tools are needed for accurate symptom discrimination. In this secondary analysis, we examined relationships between baseline assessments of pain, dyspnea, objectively measured PA, and exercise capacity in persons with COPD who previously enrolled in three PA studies. Pain was assessed with the bodily pain domain of the Veterans RAND-36 (VR-36), and dyspnea with the modified Medical Research Council (mMRC) scale. Average daily step count was assessed with the Omron HJ-720ITC or FitBit Zip pedometer, and exercise capacity with 6-minute walk test (6MWT). We also conducted a pilot neuroimaging study. Neuroimaging data were acquired on a Siemens 3-Tesla Magnetom Prisma^fit^ whole-body scanner. Analysis of variance assessed trends in daily step count and 6MWT distance across categories of co-occurring pain and dyspnea. General linear models examined relationships between cortical thickness and resting state functional connectivity (fc) with symptoms and functional status. In 373 Veterans, 98% were male with mean age 70.5± 8.3 years and FEV_1_% predicted 59 ± 21%. Compared to those with no co-occurrence of pain and dyspnea, those with co-occurrence walked 1,291–1,444 fewer steps per day and had an 80–85 m lower 6MWT distance. Ten males participated in the pilot neuroimaging study. Predominant findings were that lower cortical thickness and greater fc were associated with higher pain and dyspnea, p<0.05. Greater cortical thickness and lower fc were associated with higher daily step count and 6MWT distance, p<0.05. Regional patterns of associations differed for pain and dyspnea, suggesting that cortical thickness and fc may discriminate symptoms. Co-occurring dyspnea and pain in COPD are associated with significant reductions in PA and exercise capacity. It may be feasible for neuroimaging markers to discriminate between pain and dyspnea.

## Introduction

Chronic obstructive pulmonary disease (COPD), a major cause of global morbidity, is a leading cause of death in the United States [1]. Persons with COPD experience significant dyspnea despite optimization of medical therapy [2, 3]. In addition, pain significantly impacts health-related quality of life (HRQL) and functional ability [4–7]. Over half of persons with COPD experience chronic pain—largely musculoskeletal pain [6, 7]. The co-occurrence of dyspnea and chronic back or arthritis pain exists in 50–67% of persons examined in a Medicare database [8].

Despite the high prevalence of dyspnea and pain in patients with COPD, little is known about their relationship to physical activity (PA) and exercise [9]. Patients who suffer from both pain and dyspnea may have their perceptions of one symptom amplified or modulated by the other, particularly during PA and exercise [8, 9]. Clinically, it is often difficult to distinguish the intensity of and interference by pain clearly separated from dyspnea using self-report and questionnaires. It is important to distinguish these symptoms for optimal treatment and to minimize them as barriers to PA and exercise in COPD. Novel diagnostic tools are needed to complement self-report of symptoms for accurate assessment in persons with COPD.

Neuroimaging modalities, including structural imaging and functional imaging, may potentially distinguish neural signatures of pain and dyspnea. Structural imaging can detect variations in the amount of regional brain tissue; for example, the thickness of the cerebral cortex can be a sensitive marker of neural health and disease [10]. Resting state functional connectivity magnetic resonance imaging (fcMRI) assesses interactions among brain regions that occur in a ‘resting state,’ in the absence of sensory-motor stimulation or performance of behavioral/cognitive tasks [11]. Specifically, fc within the “default mode” network (DMN) (posterior cingulate, inferior parietal lobes, and medial frontal gyrus) shows high interconnectivity with other brain networks, making fc within the DMN a sensitive index of symptoms [12]. Differential brain structural and functional patterns of pain and dyspnea have not been previously studied in persons with COPD.

We performed a secondary analysis of baseline assessments of pain, dyspnea, objectively measured PA, and exercise capacity in a combined cohort of persons with COPD who previously enrolled in three PA studies [13–15]. We additionally performed an ancillary pilot study in a subset of participants to examine the feasibility of assessing neural signatures of pain and dyspnea using structural and functional MRI. Our aims were to: (1) characterize the prevalence of co-occurring pain and dyspnea in COPD, (2) examine the relationship between the co-occurrence of pain and dyspnea, and PA and exercise capacity, and (3) explore the feasibility of using MRI neuroimaging to distinguish between pain and dyspnea.

## Materials and methods

All data were acquired in the same manner in the three separate studies [13–15] from participants with COPD, defined as forced expiratory volume in first second (FEV_1_)/ forced vital capacity (FVC) < 0.70 or CT scan evidence of emphysema. Study 1 (n = 163) was an observational study of participants recruited from pulmonary clinics at the VA Boston Healthcare System between 2009–2011 [13]. Study 2 (n = 104) included Veterans enrolled from pulmonary clinics at VA Boston from 2012–2016 for a randomized controlled trial (RCT) (NCT01772082) comparing a web-based, pedometer-mediated PA intervention to pedometer alone [14]. Study 3 (n = 108) included people recruited from VA Boston for a RCT in which study participants were assigned to the same technology-based PA intervention or usual care (NCT02099799) [15]. All protocols were approved by the VA Boston Healthcare System Institutional Review Board, and written informed consent was obtained from each participant.

At the baseline in-person study visit, a medical history assessed demographics, medication use, and comorbidities. Information about cigarette use, supplemental oxygen use, and prior participation in pulmonary rehabilitation was obtained. Spirometry was performed according to American Thoracic Society (ATS) criteria [16, 17]. Only baseline data were used for this retrospective analysis.

### Pain and dyspnea

Pain was assessed with the Veterans SF-36 (VR-36) questionnaire using the two questions that contribute to the bodily pain domain [18]. Participants answered two questions assessing amount of pain and pain interference over the past 4 weeks: “How much bodily pain have you had during the past 4 weeks?” with responses of none, very mild, mild, moderate, severe, and very severe, and “During the past 4 weeks, how much did pain interfere with your normal work?” with responses of not at all, a little bit, moderately, quite a bit, and extremely. The overall pain domain score ranges from 0 to 100, with lower scores indicating greater pain [18]. We examined each of the response categories as a categorical variable. Two participants did not complete the VR-36; thus, we include 373 observations in this retrospective analysis.

The Brief Pain Inventory (BPI) [19] was also administered to a subgroup of participants (n = 98) in Study 3 [15] to further characterize pain. The BPI assesses pain intensity and pain interference over a recall period of one day. The BPI queries pain location, pain severity, impact of pain on daily function, pain medications, and amount of pain relief in the past 24 hours or the past week. The BPI has been validated in persons with COPD [19]. We examined summary scores, namely, the arithmetic mean of the four intensity items which measures pain intensity, and the arithmetic mean of the seven interference items which measures pain interference. We also examined responses to each question to obtain a detailed picture of pain experienced by the cohort. Scores range from 0–10, with 10 being the greatest intensity or interference [19].

Dyspnea was assessed with the modified Medical Research Council (mMRC) scale. mMRC scores range 0–4, with higher numbers representing more breathlessness [20]. The UCSD Shortness of Breath Questionnaire [21] was also administered in Study 3 [15] to further characterize dyspnea. The questionnaire has 24 items with responses ranging from 0 (not at all) to 5 (maximally or unable to do because of breathlessness). Questions ask about the amount of shortness of breath experienced while performing various activities, as well as how shortness of breath and fear of shortness of breath limit activities. We examined the summary total score.

### Physical activity and exercise capacity

At study enrollment, PA was objectively assessed with the Omron HJ-720ITC pedometer (worn for 10 or 14 days) [13, 14] or the FitBit Zip pedometer (worn for 10 days) [15], and quantified as average daily step count [22, 23]. In all cases, a sticker covered the display face of the pedometer to prevent any feedback, and participants were instructed to engage in their usual physical activities. At the baseline in-person study visit, participants performed a 6-minute walk test (6MWT) as a measure of exercise capacity, according to ATS guidelines except that a practice walk was not performed [24–26].

### Ancillary study: Neuroimaging feasibility

A convenience sample of 10 men were recruited at baseline from Study 3 [15] for the neuroimaging pilot study. Additional exclusion criteria included: known brain lesions, claustrophobia, history of seizures, diagnosis of bipolar disorder, schizophrenia, psychotic disorder, or cognitive disorder like dementia. Individuals with any contraindications for MRI, such as shrapnel, surgical medical clips, implants, or pacemakers, were also excluded. Pain and dyspnea were assessed immediately prior to the MRI.

Neuroimaging data were acquired on a Siemens 3-Tesla Magnetom Prisma^fit^ whole-body scanner with a 20-channel phased-array head coil. Two Magnetization Prepared Rapid Gradient Echo (MP-RAGE) T1-weighted structural images were acquired for each participant (TR = 2530 ms, TE = 3.35 ms, TI = 1.1 s, 200 Hz/pixel bandwith, flip angle = 7°, imaging matrix = 256 × 176, voxel size = 1 mm^3^; 3D sagittal) [27, 28]. Whole-brain resting state functional data were acquired over two runs of gradient echo echo‐planar imaging (EPI), TR/TE: 4,000/31 ms, flip angle: 90°, FOV = 128 × 128 mm, voxel size 3 × 3 × 3 mm; 90 volumes) [29, 30]. Participants were instructed to keep their eyes open and stay awake during the resting functional scanning.

T1-weighted anatomical images were automatically processed to reconstruct cortical surfaces and to segment region of interest (ROI) volumes using the FreeSurfer recon-all procedure (http://surfer.nmr.mgh.harvard.edu) [31]. The shortest distance between a vertex on the inner surface and a vertex on the outer surface was calculated to measure cortical thickness [32]. Participants were registered into a common template using a surface-based averaging technique by considering cortical folding patterns [33].

Neuroimaging data were processed for fc measures using a combination of FreeSurfer [32], AFNI [34], and FSL [35] based on the FSFAST processing stream (http://freesurfer.net/fswiki/FsFast). Scans for each subject were preprocessed using a standard stream (motion correction using six parameters, time shifting, concatenation of scans, motion regressed from time series, regression of the global mean, and the average time courses from the white matter and the ventricles, band pass filtering between 0.01 and 0.1 Hz). Data were sampled to and smoothed on the cortical surface, and data from each participant transformed to standard space using a surface‐based template fsaverage [36]. A DMN seed region in the posterior cingulate was derived from surface‐based parcellation of the cortex [31]. The bilateral superior third of the isthmus of the cingulate, as defined within each participant’s native space, was selected as the seed region [29]. Following the FreeSurfer FS-FAST processing stream, the vertex‐wise partial correlation to the DMN seed was computed and used for further group‐level analyses. Briefly, to estimate fc to the DMN seed, the mean time series of the DMN seed was first correlated with all other voxels’ time series in the brain, and then the measures were transformed onto the cortical surface and represented as vertex‐wise partial correlation (i.e., at each vertex over the cortical surface). Details can be found in FS‐FAST processing stream: http://freesurfer.net/fswiki/FsFast.

### Statistical analyses

Characteristics of the cohort and responses to the pain and dyspnea questionnaires were summarized using mean ± standard deviation or frequency (percentage) as appropriate. Normality was assessed using Shapiro Wilk tests and Q-Q plots. Analysis of variance (PROC ANOVA in SAS v9.4) assessed the trend relationships between the categorical responses to VR-36 pain amount and pain interference with dyspnea assessed by either the mMRC or UCSD SOB questionnaire. Participants were then grouped into three categories (no, some, yes) based on degree of co-occurring pain (VR-36 or BPI) and dyspnea (mMRC). Analysis of variance assessed the trend relationships between the three categories of co-occurring pain and dyspnea with the continuous variables of daily step counts (PA) and 6MWT distance (exercise capacity). Analyses were performed using SAS v9.4 (Cary, NC).

Images show an ‘inflated’ brain; the cortical surface has been computationally inflated so data in the deep sulci [dark gray, compared to gyri in light gray] are more easily visible. The colors on each brain are the result of a statistical analysis (general linear model) performed on the data at each point (~1mm spatial extent) on the cortical surface. Regions showing effects in heat color scale (red/yellow) indicate a positive association between the dependent variable (the imaging variable) and the independent variable (the clinical variables). Regions showing effects in cool color scale (light/dark blue) indicate a negative association between the dependent and independent variable. The color scale to the right of each figure translates the specific color to a p value for each point. The brightness within the heat colors (bright yellow p≤0.01/dark red p<0.05) or cool colors (bright blue p≤0.01 or dark blue p<0.05) indicates the strength of the association, with brighter areas being more statistically significant than darker areas, as shown on the color scale. General linear models examined the relationships between cortical thickness and resting state fc and pain, dyspnea, PA, and exercise capacity. Cortical thickness data were smoothed to surface-based 15 mm full-width/half-max (FWHM). We examined both hemispheres, and present findings for the right since trends were similar.

## Results and discussion

### Co-occurrence of pain and dyspnea

In the cohort of 373 Veterans with COPD, 98% were male with mean age 70.5± 8.3 years and FEV_1_% predicted 59 ± 21% (Table 1). There was a significant trend of worsening bodily pain amount (P = 0.0020; Table 2A) and interference (P<0.0001; Table 2B) associated with worsening mMRC dyspnea score. A similar significant trend was observed with the subset of 102 participants who also completed the UCSD SOB questionnaire (Table 2A and 2B).

**Table 1:** Participant characteristics.

Characteristic	
Age	70.5 ± 8.3
Sex (male)	364 (97.6%)
Race (white)	346 (92.8%)
Pack Years	62 ± 38
Angina	51 (13.7%)
Coronary Artery Disease	98 (26.%)
FEV_1_% predicted*	59 ± 21
SGRQ Total Score	40 ± 18
VR-36 Pain Domain Score	60 ± 26
Brief Pain Inventory**	
Intensity Score	3.78 ± 1.90
Interference Score	2.67 ± 2.33
mMRC^+^	
0	31 (8%)
1	134 (36%)
2	65 (17%)
3	89 (24%)
4	54 (14%)
UCSD SOB Total Score***	33 ± 22
Daily Step Counts	3,130 ± 2,333
6MWT Distance (meters)	376 ± 96

Values are mean±s.d. or frequency (percentage). n = 373 except where noted.

*n = 367.

**Based on n = 55 who endorsed pain of the n = 98 to whom the BPI was administered.

***n = 102. ^+^Does not add to 100% due to rounding.

**Table 2:** Increasing bodily pain (2a) and pain interference (2b) are associated with increasing dyspnea.

Number (%) n = 373	Bodily Pain Amount (VR-36)	Dyspnea (mMRC) mean±sd	Number (%) n = 102	Bodily Pain Amount (VR-36)	Dyspnea (UCSD SOB) mean±sd
64 (17.16%)	None	1.70 ± 1.24	19 (18.63%)	None	28.37 ± 24.89
73 (19.57%)	Very Mild	1.86 ± 1.25	23 (22.55%)	Very Mild	29.09 ± 20.69
76 (20.38%)	Mild	1.80 ± 1.05	21 (20.59%)	Mild	30.90 ± 17.49
112 (30.03%)	Moderate	2.18 ± 1.26	22 (21.57%)	Moderate	36.73 ± 21.75
42 (11.26%)	Severe	2.52 ± 1.15	15 (14.71%)	Severe	43.80 ± 20.72
6 (1.61%)	Very Severe	2.50 ± 1.64	2 (1.96%)	Very Severe	31.00 ± 36.77
P value for trend		0.0020	P value for trend		0.0057
Number (%) n = 373	Bodily Pain Interference (VR-36)	Dyspnea (mMRC) mean±sd	Number (%) n = 102	Bodily Pain Interference (VR-36)	Dyspnea (UCSD SOB) mean±sd
128 (34.32%)	Not at all	1.66 ± 1.21	38 (37.25%)	Not at all	27.32 ± 22.84
94 (25.20%)	A Little Bit	1.94 ± 1.11	26 (25.49%)	A Little Bit	28.04 ± 17.76
82 (21.98%)	Moderately	2.11 ± 1.18	22 (21.57%)	Moderately	43.32 ± 18.42
57 (15.28%)	Quite a Bit	2.58 ± 1.31	14 (13.73%)	Quite a Bit	39.36 ± 22.73
12 (3.22%)	Extremely	2.75 ± 1.06	2 (1.96%)	Extremely	56.50 ± 0.71
P value for trend		<0.0001	P value for trend		<0.0001

For any given level of pain amount and pain interference assessed by the VR-36 bodily pain questions, there was representation across the entire range of mMRC dyspnea scores (Table 3A and 3B). Only 11 participants (3%) endorsed no pain and no dyspnea (Table 3A). One hundred and sixty-four participants (44%) had high levels of pain but mild dyspnea or high levels of dyspnea and mild pain (white). One hundred and two persons (27%) had moderate to very severe pain and a mMRC dyspnea score of 2 or greater (shaded black). One hundred and seven persons (29%) had no to very mild pain and no to very little dyspnea (shaded gray). Similar proportions (white 43%, black 27%, and gray 30%) were observed for the co-occurrence of pain interference and dyspnea (Table 3B).

**Table 3:** a and b. Frequency of participants with absence of (gray) versus presence of (white and black) co-occurring pain and dyspnea.

Bodily Pain Amount (VR-36)	mMRC Dyspnea 0	1	2	3	4	Total
None	11	21	15	10	7	64
Very Mild	9	28	7	22	7	73
Mild	4	34	15	19	4	76
Moderate	5	41	19	23	24	112
Severe	1	9	9	13	10	42
Very Severe	1	1	0	2	2	6
Total	31	134	65	89	54	373
Bodily Pain Interference (VR-36)	mMRC Dyspnea 0	1	2	3	4	Total
Not at all	22	46	25	24	11	128
A Little Bit	3	43	13	27	8	94
Moderately	2	31	20	14	15	82
Quite a Bit	4	12	5	19	17	57
Extremely	0	2	2	5	3	12
Total	31	134	65	89	54	373

In the 98 participants who completed the BPI to further characterize pain, 55 participants endorsed pain—with the most frequent locations being back, knee, neck, hip, and shoulder. The average pain intensity was 3.78 ± 1.90 and pain interference was 2.67 ± 2.33, out of a maximum score of 10. Significantly, in response to the question of how much relief did you experience from treatment in the last 24 hours, the average was only 35%, with 37 (70%) endorsing 50% or less relief. Pain interference in the past 24 hours was ranked highest on walking ability 3.63 ± 3.00, compared to 2.61 ± 2.78 for general activity, 2.89 ± 2.83 for sleep, and 3.00 ± 3.19 for enjoyment of life.

Similar trends of co-occurring pain and dyspnea were observed when pain was assessed with the BPI. For pain intensity and dyspnea (Table 4A), no participant endorsed the absence of pain and dyspnea. Twenty-eight participants (51%) had high levels of pain but mild dyspnea or high levels of dyspnea and mild pain (white). Eleven persons (20%) had moderate to very severe pain and a mMRC dyspnea score of 2 or greater (shaded black). Sixteen persons (29%) had no to very mild pain and no to very little dyspnea (shaded gray). Similar proportions (white 53%, black 13%, and gray 34%) are seen for pain interference and dyspnea (Table 4B).

**Table 4:** a and 4b. Frequency of participants with absence of (gray) versus presence of (white and black) co-occurring pain and dyspnea.

Pain Intensity (BPI)	mMRC Dyspnea 0	1	2	3	4	Total
0	0	1	0	1	0	2
1,2	4	4	3	6	1	18
3,4	2	5	3	5	3	18
5,6	0	6	2	4	3	15
7,8	0	0	0	1	1	2
9,10	0	0	0	0	0	0
Total	6	16	8	17	8	55
Pain Interference (BPI)	mMRC Dyspnea 0	1	2	3	4	Total
0	5	6	3	2	0	16
1,2	1	3	3	7	3	17
3,4	0	4	2	3	3	12
5,6	0	2	0	3	2	7
7,8	0	1	0	2	0	3
9,10	0	0	0	0	0	0
Total	6	16	8	17	8	55

### Relationship between the co-occurrence of pain and dyspnea with PA and exercise capacity

The co-occurrence of pain (VR-36) and dyspnea (mMRC) was significantly associated with a trend towards lower daily step count and lower 6MWT distance (Table 5A and 5B). Compared to those with no co-occurrence of pain and dyspnea, those with co-occurrence walked, on average, 1,444 (pain amount) to 1,291 fewer steps per day (pain interference). These differences exceed the MCID of 600–1,000 for steps per day [23]. Similarly, compared to those with no co-occurrence, those with co-occurrence had, on average, an 85 m (pain amount) and 80 m (pain interference) lower 6MWT distance. These differences exceed the MCID of 35–54 m for the 6MWT distance [25, 26]. Similar significant trends were observed for pain intensity and interference assessed with the BPI (Table 6A and 6B).

**Table 5:** a and b. Co-occurring pain and dyspnea are associated with lower daily step count and 6MWT distance. n = 373.

Bodily Pain Amount (VR-36) and Dyspnea (mMRC)	Daily Step Count mean±sd	6MWT distance (meters) mean±sd
No co-occurrence, n = 107 (gray)	4,013 ± 2,467	431 ± 70
Some co-occurrence, n = 164 (white)	2,902 ± 2,219	358 ± 99
Yes co-occurrence, n = 102 (black)	2,569 ± 2,117	346 ± 91
P value for trend	<0.0001	<0.0001
Bodily Pain Interference (VR-36) and Dyspnea (mMRC)	Daily Step Count mean±sd	6MWT distance (meters) mean±sd
No co-occurrence, n = 114 (gray)	3,877 ± 2,460	424 ± 76
Some co-occurrence, n = 159 (white)	2,936 ± 2,238	362 ± 95
Yes co-occurrence, n = 100 (black)	2,586 ± 2,133	344 ± 97
P value for trend	<0.0001	<0.0001

**Table 6:** a and 6b. Co-occurring pain and dyspnea are associated with lower daily step count and 6MWT distance. n = 55.

Pain Intensity (BPI) and Dyspnea (mMRC)	Daily Step Count* mean±sd	6MWT distance (meters) mean±sd
No co-occurrence (gray)	5,536 ± 3,876 n = 20	426 ± 83 n = 22
Some co-occurrence (white)	3,153 ± 2,410 n = 20	344 ± 111 n = 22
Yes co-occurrence (black)	2,516 ± 1,180 n = 11	348 ± 78 n = 11
P value for trend	0.0105	0.0128
Pain Interference (BPI) and Dyspnea (mMRC)	Daily Step Count* mean±sd	6MWT distance (meters) mean±sd
No co-occurrence (gray)	5,536 ± 3,876 n = 20	426 ± 83 n = 22
Some co-occurrence (white)	2,978 ± 2,254 n = 24	344 ± 104 n = 26
Yes co-occurrence (black)	2,752 ± 1,294 n = 7	353 ± 89 n = 7
P value for trend	0.0123	0.0125

*n = 51 as 4 participants did not have daily step count.

### Pilot neuroimaging feasibility study

The ten participants who participated in the neuroimaging pilot study had mean age 74±4 years and FEV_1_ % predicted 68±20%. At the last study visit, bodily pain amount assessed by the VR-36 were none (n = 2), very mild (n = 4), mild (n = 3), and moderate (n = 1). At the time of the MRI, pain was assessed with the BPI and dyspnea with the mMRC scale. Dyspnea responses were 0 (n = 4), 1 (n = 3), 2 (n = 1), 3 (n = 1), and 4 (n = 1). Three of the 10 participants self-reported both pain and dyspnea at the time of imaging. At baseline, mean daily step count was 3,466±988 and 6MWT distance was 443±60 m.

**Fig 1:**
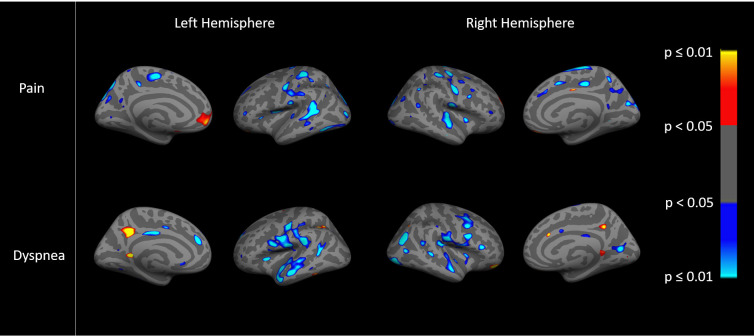
Associations between cortical thickness with pain (top) and dyspnea (bottom).

Associations between cortical thickness with pain and dyspnea are displayed in Fig 1. The predominant effects demonstrate negative associations (light/dark blue) where lower cortical thickness was associated with higher pain scores, p<0.05 (Fig 1). Similarly, lower cortical thickness was associated with higher mMRC dyspnea scores, p<0.05 (Fig 1). In addition, there were small regions with positive associations (red/yellow) between greater cortical thickness and higher pain or dyspnea p<0.05. Fig 1 appears to show different regional patterns of associations for pain and dyspnea, suggesting that cortical thickness may discriminate between the relative presence of the two symptoms.

**Fig 2:**
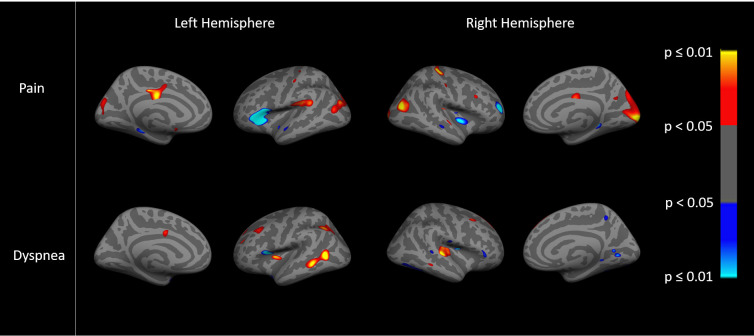
Associations between DMN functional connectivity with pain (top) and dyspnea (bottom).

Functional connectivity to the isthmus cingulate within the DMN for participants who experienced pain or dyspnea is shown in Fig 2. Although the predominant result was that greater fc was positively associated (red/yellow) with higher pain and dyspnea, there were also regions with negative (light/dark blue) associations such that greater fc was associated with lower pain and dyspnea, p<0.05 (Fig 2). Fig 2 also appears to show different regional patterns of associations suggesting that fc may discriminate between the relative presence of pain and dyspnea.

**Fig 3:**
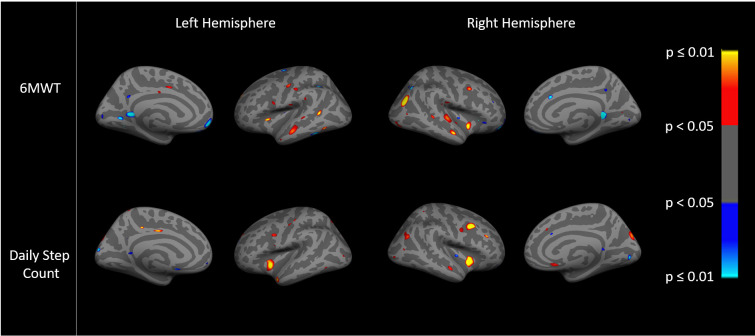
Association between cortical thickness with exercise capacity (6MWT distance) (top) and physical activity (daily step count) (bottom).

Whole brain vertex-wise associations between cortical thickness and PA as measured by daily step count are shown in Fig 3. The predominant associations were such that greater cortical thickness was positively associated with higher daily step count, p<0.05. Greater cortical thickness was also positively associated with higher exercise capacity measured by the 6MWT distance, p<0.05 (Fig 3). In addition, there were significant regions of interest with negative associations (light/dark blue) between greater cortical thickness and lower PA or 6MWT distance, p<0.05 (Fig 3). The patterns of association with cortical thickness appeared grossly similar for PA compared to exercise capacity (Fig 3).

**Fig 4:**
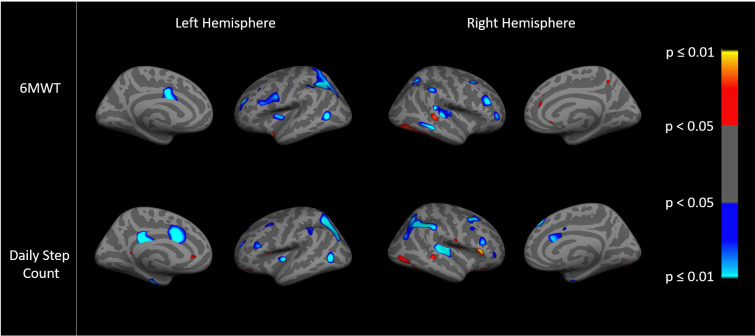
Association between DMN functional connectivity with exercise capacity (6MWT distance) (top) and physical activity (daily step count) (bottom).

In the analysis of fc within the DMN, the predominant associations were such that lower fc was negatively associated (light/dark blue) with higher daily step count and higher 6MWT distance, p<0.05 (Fig 4). In addition, there were significant regions of interest with positive associations (red/yellow) between greater fc and higher PA or 6MWT distance, p<0.05 (Fig 4). The patterns of association with fc appeared grossly similar for PA compared to exercise capacity (Fig 4).

We show that persons with COPD have a high prevalence of co-occurring dyspnea and pain, and that the co-occurrence of symptoms is associated with significant reductions in PA and exercise capacity. In addition, it may be feasible for MRI cortical morphometry and resting state fc to provide novel neural indices associated with pain and dyspnea. That regional patterns of associations in cortical thickness and fc differ for pain and dyspnea, while patterns are similar for PA and exercise capacity, which are closely related constructs, suggests that unique neuroimaging patterns may potentially distinguish between pain and dyspnea.

Our results confirm previous findings that persons with COPD have a high prevalence of co-occurring pain and dyspnea [5–8]. However, what is novel is that we studied persons with COPD who voluntarily participated in three separate PA studies, two of which involved a PA intervention. When counseling patients with COPD to exercise, it is important to note that, even in those ready to engage in exercise, there is a prevalence of co-occurring dyspnea and pain higher than what has been previously reported from a Medicare database of all-comers with COPD [8]. Symptoms should be routinely assessed and treated in clinical care.

We also demonstrate that those with co-occurring symptoms have significantly reduced functional status. These results highlight that COPD patients are impacted in their community-based walking by poorly-controlled pain and dyspnea. Optimal treatment of symptoms alone may increase daily PA, independent of participation in active interventions such as pulmonary rehabilitation or PA programs. It is unclear whether patients with COPD with pain and dyspnea refrain from exercise or if they use PA and exercise for symptom relief. It is telling that this cohort of persons with COPD voluntarily agreed to participate in PA research studies despite our findings that those with co-occurring symptoms have significantly reduced functional status.

It is challenging for healthcare providers to manage the symptoms of pain and dyspnea. As demonstrated by our significant findings that pain levels are positively associated with dyspnea levels, patients who experience pain may have the pain heightened by worsening dyspnea. Narcotic overuse for pain can lead to respiratory suppression or opioid dependence. Similarly, a patient’s report of dyspnea, amplified by worsening pain, may lead to unnecessary testing for etiologies of dyspnea and undertreatment of pain. Novel diagnostic tools are needed to complement self-reported symptoms to try to accurately distinguish pain from dyspnea.

We demonstrate feasibility in using objective central biomarkers (cortical thickness and fc) to correlate with subjective symptoms of pain and dyspnea. The different patterns of association between cortical thickness and symptoms in this pilot sample suggest that there are distinctive cortical morphometric patterns associated with each symptom. It is interesting that lower cortical morphometrics were negatively associated with higher levels of the symptoms of pain and dyspnea, while greater fc was positively associated with higher pain and higher dyspnea. Patient self-report, along with neuroimaging, may allow for more accurate assessment and discrimination of dyspnea and pain.

Greater cortical thickness was positively associated with higher levels of two measures of functional status—PA and exercise capacity, while negative relationships between greater fc with lower daily step count and lower 6MWT distance were observed. The significance of the positive versus negative relationships between cortical thickness and fc with daily step count and 6MWT distance is unclear and warrants further evaluation in a larger cohort. We acknowledge that our pilot results are from a very small cohort of persons with COPD. Nevertheless, we have extended the limited literature currently available that examines resting state fc, brain activity, or the DMN in persons with COPD [37–41].

Neuroimaging of pain and dyspnea is in its infancy [42, 43]. In healthy humans, resting state fc, between the anterior insular cortex and brainstem periaqueductal gray, has been shown to play an important role in pain perception [44]. These resting communications are altered in older adults with chronic musculoskeletal pain who show greater fc between the posterior cingulate and left insula, superior temporal gyrus, and cerebellum [45]. Furthermore, “dyspnea is not a carbon copy of pain [42, 43].” Most studies of neuroimaging and dyspnea to date have focused on acute dyspnea elicited in healthy volunteers. Only two studies have examined dyspnea in patients with COPD, and characterized its association with resting state fc in the anterior insular cortex, anterior cingulate cortex, prefrontal cortex, and posterior insular cortex [37, 38]. Even less is known about cortical morphometry or fc in persons with COPD who experience co-occurring chronic pain and dyspnea [43]. To our knowledge this is the first study to examine neuroimaging in persons with COPD with co-occurring dyspnea and pain. That regional patterns of associations in cortical thickness and fc differ for pain and dyspnea, while patterns are similar for PA and exercise capacity, which are closely related constructs, suggests that unique neuroimaging patterns may potentially distinguish between pain and dyspnea.

A major strength of this retrospective analysis is the large, well-characterized cohort of persons with COPD in whom we assessed both pain and dyspnea concurrently and objectively measured PA. We acknowledge that the BPI and UCSD SOB questionnaires were assessed in only a subgroup of participants, but their results are the same as those for the VR-36 pain questions and the mMRC dyspnea scale which were assessed in the entire cohort. Types and location of pain, such as thoracic or musculoskeletal, were not assessed. Our results should be interpreted knowing that there were participants who self-reported angina and history of coronary artery disease, which may contribute to dyspnea and pain. We examined only a single functional network selected due to prior demonstrations of the regions of the DMN supporting critical higher cognitive and behavioral functions. However, it is possible that other networks may be linked to symptoms of pain and dyspnea, and we will examine this in expanded future work. There are strong implications for future translation into clinical practice to potentially (1) accurately assess baseline and relative changes in pain and dyspnea in response to exercise rehabilitation, and (2) inform the design of interventions to improve treatment of these symptoms. Future work in larger cohorts will examine the unique patterns of cortical thickness and fc as novel biomarkers to assess underlying mechanisms of symptom perception and understand the unique contributions of pain and dyspnea to a COPD patient’s symptom complex, ultimately for accurate diagnosis and clinical management.

## Conclusions

Persons with COPD have a high prevalence of co-occurring dyspnea and pain, and the co-occurrence of symptoms is associated with significant reductions in PA and exercise capacity. In addition, it may be feasible for MRI cortical morphometry and resting state fc to provide novel neural indices associated with pain and dyspnea. Unique neuroimaging patterns may potentially distinguish pain from dyspnea.
